# High Ocular Disease Burden and Increased Referral Needs in Patients with Chronic Kidney Disease: A Step Toward Personalized Care

**DOI:** 10.3390/jpm15050204

**Published:** 2025-05-19

**Authors:** Yulia Liem, Pavitra Thyagarajan, Miao Li Chee, Cynthia Ciwei Lim, Boon Wee Teo, Charumathi Sabanayagam

**Affiliations:** 1 Singapore Eye Research Institute, Singapore National Eye Centre, Singapore 169856, Singapore; yulia.liem@seri.com.sg (Y.L.); e0498207@u.nus.edu (P.T.); chee.miao.li@seri.com.sg (M.L.C.); 2 Department of Renal Medicine, Singapore General Hospital, Singapore 169856, Singapore; cynthia.lim.c.w@singhealth.com.sg; 3 Department of Medicine, Yong Loo Lin School of Medicine, National University of Singapore, Singapore 117597, Singapore; mdctbw@nus.edu.sg; 4 Division of Nephrology, Department of Medicine, University Medicine Cluster, National University Health System, Singapore 119228, Singapore; 5 Ophthalmology and Visual Sciences Academic Clinical Programme, Duke-NUS Medical School, Singapore 169857, Singapore

**Keywords:** chronic kidney disease, eye diseases, prevalence, referral

## Abstract

**Background/Objectives:** To evaluate the prevalence of eye diseases in patients with confirmed chronic kidney disease (CKD) and their referral patterns to ophthalmologists, with the aim of informing personalized screening and referral strategies. **Methods:** This study involved 528 CKD patients from a tertiary hospital’s outpatient renal clinics in Singapore, with CKD defined as an estimated glomerular filtration rate (eGFR) < 60 mL/min/1.73 m^2^. Retinal photographs from each dilated eye were graded for the presence of diabetic retinopathy (DR) and other eye diseases by professional graders. Patients with significant eye conditions were referred to ophthalmologists based on severity and urgency, categorized as urgent (same day or within 24 h), semi-urgent (within 1–2 weeks), fast-track (within 1–3 months), or annual referrals. **Results:** More than half of the CKD patients (53.7%) had some form of eye disease; 20% were diagnosed with DR, and 29% required fast-track referrals. Of the 251 patients with diabetes, 67% adhered to annual follow-ups; however, despite this regular monitoring, over half required fast-track referrals for severe eye conditions. Among the 167 non-diabetic CKD patients, nearly a third (31%) were on follow-up, with 7.8% requiring fast-track referrals. Notably, 11% of those not on follow-up also needed fast-track referrals. Seven non-diabetic and ten diabetic patients required urgent referral due to critical conditions such as pseudo-holes, impending occlusions, and disc swelling. **Conclusions:** These findings underscore the high prevalence and severe nature of eye diseases in CKD patients, even those who are under regular annual follow-up. Integrating systematic eye screening into CKD care supports personalized medicine by enabling early detection and tailored interventions, ultimately improving both visual and overall patient outcomes.

## 1. Introduction

Chronic kidney disease (CKD) is a growing health burden worldwide, associated with an increased risk of cardiovascular disease [1], renal failure that requires dialysis and/or transplant, premature death, and a decrease in the quality of life [2]. CKD, defined as an estimated glomerular filtration rate (eGFR) < 60 mL/min/1.73 m^2^, is estimated to have affected ~10% of the population worldwide in 2022 [3]. It is classified into five stages, with stages G3–G5 reflecting moderate to severe disease and a higher risk of complications. The incidence of CKD is expected to rise further due to population aging and the increasing prevalence of diabetes and hypertension.

Globally, Singapore ranks fourth for existing kidney failure cases, while it ranks first for diabetes-induced kidney failure [4]. With an aging population and the rising prevalence of diabetes and hypertension, vision impairment (VI) has also become a major public health issue worldwide, affecting at least one billion people and disproportionately affecting those in low- and middle-income countries [5]. While cataracts and refractive errors are the leading causes of VI, a variety of other disorders, including diabetic retinopathy (DR), age-related macular degeneration (AMD), and glaucoma, also play an important role in causing VI and blindness [6,7,8,9]. Ensuring early detection, adherence to referrals, and timely interventions is paramount to reducing the burden of VI.

Emerging evidence supports the role of personalized medicine in managing CKD, where patient-specific characteristics, including comorbidities, disease progression, and functional status, can guide targeted interventions [10,11]. This approach is equally critical for CKD patients with co-existing eye diseases. The primary goal of eye screening in CKD patients is the identification of eyes at high risk for disease progression and visual loss. Timely detection and referral are especially crucial for conditions such as proliferative diabetic retinopathy (PDR), central retinal arterial occlusion (CRAO), pseudo-holes, and retinal detachment, which are often asymptomatic but can result in severe vision loss or blindness if left untreated.

The eye and the kidney share similar physiological and pathogenic pathways [12,13], as well as common risk factors. Several studies, including our own, have consistently demonstrated an association between CKD and ocular conditions, highlighting a heightened prevalence of VI among CKD patients [14,15,16,17]. Our earlier study of a middle-aged, population-based cohort found that CKD participants had nearly three times the prevalence of VI and 1.6 times the prevalence of any ocular disease compared to those without CKD [17]. However, referral patterns were not evaluated in previous studies. Information on the referral patterns may help clarify the eye care needs among individuals with CKD and potentially identify gaps or opportunities to improve the effectiveness of current screening practices in detecting and managing eye conditions.

In this study, we aimed to assess the presence and severity of eye diseases using dilated fundus images in a CKD cohort and to evaluate referral frequencies and patterns.

## 2. Materials and Methods

### 2.1. Study Population

We included 724 patients aged ≥ 40 years with clinically confirmed CKD, recruited from the Division of Nephrology outpatient clinics at the National University Hospital (NUH) between 2013 and 2017, as part of the Retinal Imaging in Renal Disease (IRED) Study. The inclusion criteria were: (1) age ≥ 40 years; (2) CKD clinically confirmed by a nephrologist; and (3) outpatient status. The exclusion criteria were: (1) unwillingness to participate in the study; and (2) individuals with limb amputations or those who used a wheelchair. We defined CKD as an eGFR of <60 mL/min per 1.73 m^2^ from serum creatinine, corresponding to CKD Stage G3 and above. Albuminuria was assessed by urine albumin-to-creatinine ratio (UACR), expressed in mg/g. Creatinine and UACR were assessed using standard laboratory methods, as described in the study by Teo et al., 2014 [18].

In the current analysis, we included only individuals with an eGFR < 60 mL/min/1.73 m^2^, thereby excluding those with early-stage CKD (Stages G1 and G2: eGFR ≥ 60) who may have had kidney damage based on UACR, as UACR data were not consistently available for this group. Additionally, an eGFR < 60 mL/min/1.73 m^2^ was chosen as the threshold, since it marks the stage at which complications and adverse outcomes become more likely. After further excluding participants with poor-quality retinal images (n = 37) and those with missing eGFR data (n = 10), the final sample size for the present study was 528. This study was conducted in accordance with the tenets of the Declaration of Helsinki, with ethics approval obtained from the NUH and the SingHealth Institutional Review Board (IRB). Informed consent was obtained from all participants.

### 2.2. Retinal Photography and Eye Diseases

After dilating the pupils with mydriatic drops, two-field (optic disc-centred and macula-centred) retinal fundus photograph images of both eyes were taken using a digital retinal camera (Canon CR-DGi non-mydriatic) by a trained research coordinator. The images were assessed for any fundus abnormalities—including the presence of retinopathy, age-related macular degeneration (AMD), glaucoma suspect, and other serious eye diseases such as retinal vein occlusion—by trained graders at the Singapore National Eye Centre, following a standard protocol. If the fundus image quality was poor and unsuitable for grading, patients were referred to ophthalmologists at specialist centres. Retinopathy was defined as the presence of microaneurysms, hemorrhages, cotton wool spots, hard exudates, intraretinal microvascular abnormalities, or neovascularization. DR was defined as the presence of any retinopathy lesions in patients with diabetes. Other eye diseases were identified based on specific lesions observed in the retinal images, e.g., exudates within 1 disc diopter of fovea or a group of exudates within 2 disc diopters of fovea (maculopathy); macular pigment abnormalities or geographic atrophy (AMD); media opacity (cataracts); and a cup-to-disc ratio ≥ 0.65, disc asymmetry ≥ 0.2, disc hemorrhage, rim thinning (glaucoma suspect), etc. Other eye diseases included cataracts, retinal detachment, pseudo-holes, retinal emboli, disc swelling, macula hemorrhage, etc. Any ocular disease was defined as having retinopathy or any of the other age-related eye diseases in retinal images.

### 2.3. Assessment of Relevant Clinical Data

Information on patients was collected through detailed history, clinical examination, and laboratory investigations. Relevant patient data collected included patient demographics (age, sex, ethnicity), comorbidities (diabetes mellitus, hypertension, anemia, and dyslipidemia), medication history, and regular eye examination status. Laboratory examination included testing blood for creatinine, glycated hemoglobin (HbA1c), fasting glucose, lipid profile, and UACR. Clinical examination included assessment of height, weight, and blood pressure.

### 2.4. Outcome Definitions

The primary outcomes of this study were the rates of referrals to ophthalmologists within a three-month timeframe and the referral patterns among CKD patients. Referral guidelines were based on established standards that stipulate the timeframes of referral to ophthalmologists based on the degree of VI and the severity of eye disease [19]. The referral timeframes were categorized as: (1) urgent—same day or within 24 h; (2) semi-urgent—within 1–2 weeks; (3) fast-track—within 1–3 months; and (4) annual (Table 1).

This framework provides a structured approach to ensure timely and appropriate care for patients. Patients with VI or vision-threatening eye conditions identified through retinal photography were referred to ophthalmologists based on the urgency and severity of their condition. Referral urgency was determined not only by the diagnostic label but also by disease activity, severity, and the risk of vision loss as observed on retinal imaging. Therefore, the same condition could warrant different referral timelines depending on its clinical presentation. For example, PDR was referred urgently when presenting with neovascularization of the disc, vitreous hemorrhage, or signs of tractional retinal detachment. In contrast, stable PDR with maculopathy but without acute complications was referred semi-urgently, within 1–2 weeks. Similarly, when patients had AMD of differing severity in each eye (e.g., late AMD in one eye and early in the other), referral classification was determined based on the more advanced stage. Among those with late AMD, referral timing was further stratified based on specific clinical features: patients with pigment epithelial detachment or choroidal neovascular membrane were assigned a referral within 1 week, while those with geographic atrophy involving the central 2 disc diameters or a subretinal fibrous scar were assigned a referral within 1 month. Our study applied a simplified classification of “early” and “late” AMD to ensure consistency across grading. As a result, intermediate AMD cases were grouped with early AMD, which may have influenced the timing of referrals for late AMD.

Urgent referrals are crucial for conditions such as PDR, late AMD, disc swelling, and retinal artery or vein occlusion. Referrals within 1–2 weeks are recommended for conditions such as retinal emboli, central serous retinopathy, and impending retinal occlusions. Fast-track referrals are made for moderate DR, glaucoma suspects, epiretinal membranes (ERM), and media opacity with poor visual acuity. Patients with ungradable images were also given fast-track referrals. Other eye conditions requiring non-urgent annual rescreening included mild DR, early/intermediate AMD, and media opacity with better VA. The secondary outcome of this study was to investigate the prevalence of eye diseases and the association of risk factors with any ocular disease, and the presence of specific ocular diseases including AMD, cataracts, and DR.

### 2.5. Statistical Analyses

All statistical analyses were performed using R (Version 4.3.1). Comparisons of systemic and demographic characteristics between patients with and without ocular diseases, as well as between those with and without retinopathy, were conducted using Student’s *t*-test and the chi-square test for continuous and categorical variables, respectively. Referral frequency, patterns, and reasons for referral were identified. We also stratified patients by diabetes status and whether they were scheduled for follow-up with ophthalmologists. As the prevalence of AMD, cataracts, and DR was higher in our CKD cohort compared to other ocular conditions, we further investigated the association of risk factors with major ocular diseases such as DR and AMD using logistic regression analyses. Established risk factors, such as age, sex, body mass index (BMI), presence of diabetes, and hypertension, were adjusted for in the multivariable logistic regression model. For DR (including only those with diabetes), we also adjusted for HbA1c level.

## 3. Results

As shown in Table 2, compared to patients without any ocular disease, those with ocular disease were more likely to be older, have higher systolic blood pressure and HbA1c levels, and lower levels of eGFR. Both groups had a similar ethnic distribution, with Chinese as the majority, followed by Malays, Indians, and others, with no significant differences observed.

Of the 528 participants, 187 (35%) required referral for ocular conditions and 154 (29%) were recommended for annual rescreening for conditions such as mild NPDR or early AMD, as shown in Table 1. The remaining 187 (35%) participants did not require any referral. Among those referred, six received urgent referrals for conditions such as CRAO, macular hole, PDR, retinal detachment, and retinal emboli. An additional 30 participants were referred within 1–2 weeks (semi-urgent), the majority of which were for PDR at 63% (n = 19), followed by late AMD at 20% (n = 6). Within the three-month referral group, 29% (n = 151) had fast-track referral. Among these, two cases had ungradable images, while 17 cases were referred for monitoring due to “treated stable DR”. When stratified into CKD stages, referral rates for <3 months (n = 161), referral rates increased with CKD progression from, 43% in Stage G3, to 58% in G4, and 72% in G5.

**Figure 1:**
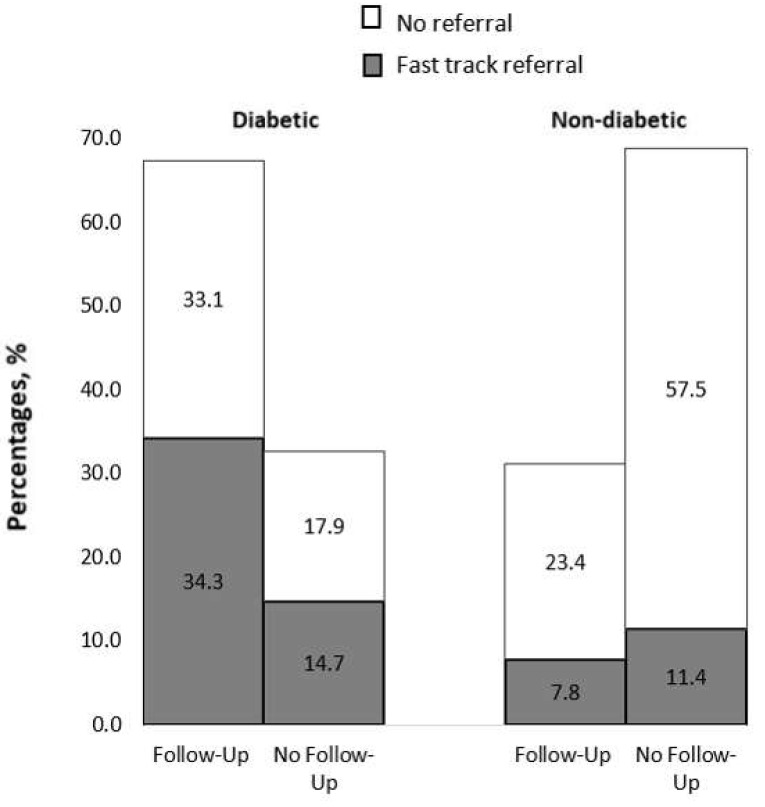
Percentages of routine monitoring among CKD patients, categorized by diabetic (n = 251) and non-diabetic (n = 167) status.

When stratified by diabetes status and follow-up status when available (n = 418) (Figure 1), 67.4% of the 251 participants with diabetes were on annual follow-up. However, 51% of those referred to annual follow-up had to be fast-track referred for conditions like referable DR, glaucoma/glaucoma suspect, ERM, AMD, etc. Of the 167 non-diabetic participants, 31.1% were on follow-up, among whom 7.8% required fast-track referral. Of the non-diabetic participants not on eye follow-up (69% in total), 11% required fast-track referral. Six of the non-diabetic and 12 of the diabetic patients required urgent referral within one week for conditions such as pseudo-holes, impending occlusions, disc swelling, etc.

### Prevalence of Eye Diseases

**Figure 2:**
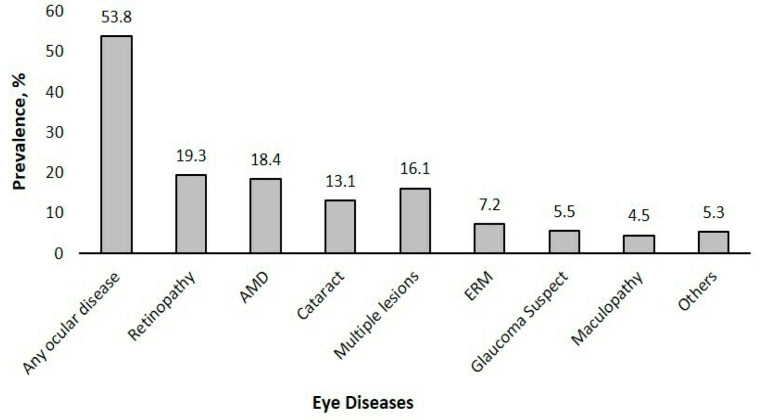
Prevalence of eye diseases (n = 528). Abbreviations: AMD, age-related macular degeneration; ERM, epiretinal membrane; Others include retinal detachment, corneal diseases, or optic neuropathy. Retinopathy includes DR. Data are presented in percentages.

Among the 528 CKD patients, 53.8% had any ocular disease, 19.3% had any retinopathy, 18.4% had AMD, 16.1% had multiple lesions, 13.1% had cataracts, 7.2% had ERM, 5.5% were glaucoma suspects, and 5.3% had other eye diseases such as retinal detachment, corneal diseases, or optic neuropathy (Figure 2). Among those with diabetes (n = 308), 27.9% had any DR, with 11.4% affected by mild NPDR, 9.7% by moderate NPDR, 1.3% by severe NPDR, and 5.5% by PDR.

For any ocular disease and AMD, in multivariable models, age, BMI, and diabetes exhibited significant associations. For DR, age showed an inverse association, while HbA1c showed a positive association (Table 3).

## 4. Discussion

We found that more than half of the CKD patients (53.8%) had any ocular disease, with conditions like DR, cataracts, and AMD being the most common. Additionally, 35% required referrals for ocular conditions. Referral rates increased with CKD progression, with a higher proportion of fast-track referrals observed in advanced CKD stages, highlighting the need for regular eye screening in this population. CKD patients with diabetes had a higher rate of follow-up, reflecting the mandate for annual eye screening for all diabetic patients (Figure 1). The findings in this study align with the growing emphasis on personalized medicine, which supports stratifying care intensity and screening needs based on disease severity and individual risk factors such as diabetes status, HbA1c levels, and CKD stage. Similar to emerging integrated care models, like the Kidney–Heart Outpatient Service [20], a collaborative approach between nephrology and ophthalmology could be developed to incorporate routine eye assessments into CKD management. This would help address the high burden of undetected ocular disease in this population.

In this study, six urgent referrals required immediate review by an ophthalmologist. Specifically, late AMD, PDR, and other conditions such as central retinal arterial occlusion, retinal detachment, or disc swelling required urgent referrals within a week. Other studies, including our earlier study, have shown that the prevalence and severity of eye conditions (Table 4), such as AMD and DR, are higher in CKD patients compared to non-CKD patients [17]. However, this study also identified other ocular conditions requiring semi-urgent referral within 1–2 weeks, many of which frequently co-existed with additional eye diseases. A recent study in India by Mohan et al. (2024) showed that other ocular conditions, such as CRAO and ERM, were commonly detected in CKD patients [21]. Several studies have also shown that CRAO is associated with end-stage kidney disease [22,23,24]. Another ocular screening study conducted by Michaud et al. in Canada, which used refractive-based symptoms, detected asymptomatic eye conditions, some of which required urgent referral in the absence of associated symptoms or prior awareness of the disease’s presence. This finding suggests that undiagnosed conditions may remain unnoticed without routine eye check-ups [25]. Early detection of ocular conditions in CKD patients enables timely referral and intervention, reducing the risk of vision loss. While urgent referral guidelines vary across healthcare systems, a consensus exists that all PDR cases require urgent referral. In this study, other ocular conditions requiring urgent referral were detected incidentally during screening and often co-existed with other eye conditions, reinforcing the need for regular eye examinations in CKD populations.

In this study, cases requiring fast-track referral included glaucoma suspects, cataracts, moderate NPDR, and late AMD cases. Additionally, patients with “treated stable DR” were referred despite appearing stable at the time of screening, for several possible reasons. Treated DR necessitates long-term monitoring, as the risk of progression remains even after interventions such as anti-vascular endothelial growth factor injections or panretinal photocoagulation [29]. The presence of residual lesions like hard exudates or new clinical signs such as macular edema or microaneurysms may not be clearly captured in screening images, prompting cautious referral [30]. Furthermore, patients with a history of DR treatment may be referred to ensure that they are being monitored. Fast-track referral is critical to ensure that any early signs of disease progression can be addressed promptly, thereby minimizing the risk of vision loss. The Blue Mountain eye study showed that moderate CKD patients had a threefold increased risk of developing early AMD within five years compared to those with mild or no CKD, while data from Korea indicated a 33% higher risk of end-stage kidney disease among AMD patients with visual disabilities [28,31]. Positive associations have also been identified between CKD and open-angle glaucoma, highlighting the interrelationship between these conditions [32]. The increased risk may be attributed to shared mechanisms such as oxidative stress, inflammation, and renin–angiotensin system dysfunctions. In glaucoma, oxidative stress is recognized for its detrimental effects on retinal cells, similar to its involvement in CKD, where it facilitates renal fibrosis and disease advancement [33,34]. Similarly, the renin–angiotensin system may influence glaucoma by modulating intraocular pressure control [35]. Identifying these common pathways suggests that managing one condition may affect the other, underscoring the need for integrated care strategies. Tailoring referral needs, follow-up schedules, imaging modalities, and treatment approaches based on CKD severity and individual ocular risk profiles is critical to preventing irreversible vision loss. Such personalized approaches not only address each patient’s unique clinical needs but also improve treatment adherence and outcomes. Given these interactions and elevated risk profiles, routine eye screenings should be incorporated into CKD management to facilitate early detection, reduce the risk of vision-threatening complications, and better address the complex needs of this population. Eye diseases were highly prevalent in both diabetic and non-diabetic CKD patients. Our study findings indicate that while 50% of individuals with both diabetes and CKD received referrals within three months, 20% of non-diabetic CKD patients also required referrals, including 7.8% cases classified as urgent. Yet, routine eye screening is not currently standard practice for CKD patients. Diabetic patients receive annual retinal screenings for referable DR, but no established guidelines exist for eye screening in non-diabetic CKD patients, highlighting a critical gap in care. When stratified by follow-up status, we found that nearly one-third of patients with diabetes did not attend annual retinal screenings. Of those who attended annual retinal screenings, more than 50% still required referrals to ophthalmologists within three months, and 10 of these patients required urgent referrals for conditions such as pseudo-holes, impending occlusions, and disc swelling. These findings emphasize that, despite annual DR screening for diabetic patients, those with CKD may experience accelerated progression of eye conditions. This underscores the critical need for frequent and comprehensive eye screenings for CKD patients with diabetes to enable prompt detection and management of severe eye diseases, thereby protecting their visual health. Among the risk factors, we found that age, diabetes, and HbA1c were associated with ocular diseases in CKD patients, suggesting that both CKD and ocular conditions are clinical outcomes of underlying microvascular disease. Growing evidence of microangiopathy, inflammation, and oxidative stress, along with shared risk factors like age, smoking, hypertension, diabetes, high cholesterol, and obesity, suggests that the eye and kidney follow similar pathogenic pathways. Advanced glycation end products form in response to oxidative stress or elevated blood sugar levels [36]. In DR, advanced glycation end products promote retinal pericyte apoptosis, compromise the integrity of the inner blood-retinal barrier, and increase the production of pro-inflammatory cytokines inside retinal vasculature and neuroglial cells [37]. Studies have also reported that elevated serum levels of advanced glycation end products bind to receptors on podocytes and endothelial cells, triggering cell death and leading to increased pro-inflammatory markers in CKD patients [38,39]. Early intervention and management of existing conditions are crucial to preserving both renal function and ocular health in CKD patients.

The strength of this study lies in its utilization of dilated pupil examinations and retinal imaging, assessed by professional graders, to effectively screen for significant ocular diseases and track referrals to ophthalmologists. Additionally, unlike many previous studies [40,41,42], including our own earlier work [17], which assessed CKD based on a single creatinine measurement and risked misclassification, this study included only confirmed CKD patients, ensuring greater accuracy. A limitation of this study is that we did not track referral uptake by patients or analyze outcomes based on dialysis status. Given that hemodialysis patients have a higher prevalence of vision-threatening retinal vascular complications, such as DR, hypertensive retinopathy, and vessel occlusions [43], future studies should evaluate these associations in relation to dialysis status.

Given the worldwide rise in CKD prevalence and the high burden of ocular conditions in this vulnerable population, our findings emphasize the critical role of incorporating regular ocular examinations into CKD care. The absence of eye screening guidelines for CKD patients is concerning, especially since many vision-threatening conditions are asymptomatic and may be present even in patients already receiving annual diabetic eye care. Integrating retinal photography into nephrology care could support earlier detection and timely intervention, which aligns with personalized medicine by tailoring care based on CKD stages, diabetic status, and referral needs. This study represents a significant step forward for personalized care for CKD patients. Additionally, eye screening may prove cost-effective by preventing advanced eye disease and promoting health equity through improved access to comprehensive care, ultimately supporting better visual outcomes for CKD patients.

## Figures and Tables

**Table 1:** Classification of referrals based on urgency and ocular conditions (n = 341).

Referral	Number of Cases	Causes
Urgent (immediate)	6	CRAO, macular hole, PDR, retinal detachment, retinal emboli
Semi-urgent (1–2 weeks)	30	Central Serous Retinopathy, collaterals with impending occlusion/disc collaterals, CRVO, late AMD, PDR with maculopathy, pseudohole due to ERM
Fast-track (1–3 months)	151	Cataracts, glaucoma suspect, late AMD, maculopathy, mild NPDR with maculopathy, moderate NPDR, ungradable, treated stable DR
Routine (Annually)	154	Early AMD, mild NPDR, myopic degeneration, presence of asteroid hyalosis

Abbreviations: AMD: age-related macular degeneration; CRAO: central retinal artery occlusion; CRVO: central retinal vein occlusion; ERM: epiretinal membrane; NPDR: non-proliferative diabetic retinopathy; PDR: proliferative diabetic retinopathy.

**Table 2:** Characteristics of CKD patients stratified by presence of any ocular disease.

Characteristics	Overall (n = 528)	No Ocular Disease (n = 244)	Ocular Disease (n = 284)	*p*-Value
Age, years	63.74 ± 10.43	61.2 ± 9.80	65.9 ± 10.5	<0.001 *
Sex				
Male	338 (64.0%)	150 (61.5%)	188 (66.2%)	0.3
Female	190 (36.0%)	94 (38.5%)	96 (33.8%)	
Ethnicity				
Chinese	336 (63.6%)	153 (62.7%)	183 (64.4%)	0.9
Malay	117 (22.2%)	54 (22.1%)	63 (22.2%)	
Indian	31 (5.9%)	14 (5.7%)	17 (6.0%)	
Others	44 (8.3%)	23 (9.4%)	21 (7.4%)	
Diabetes status				
No	220 (41.7%)	127 (52.0%)	93 (32.7%)	<0.001 *
Yes	308 (58.3%)	117 (48.0%)	191 (67.3%)	
Hypertension status				
No	48 (9.1%)	27 (11.1%)	21 (7.4%)	0.2
Yes	480 (90.9%)	217 (88.9%)	263 (92.6%)	
HbA1c, %	26.99 ± 5.14	6.6 ± 1.3	7.1 ± 1.7	<0.001 *
BMI, Kg/m^2^	6.88 ± 1.56	27.3 ± 5.3	26.7 ± 5.0	0.2
Systolic blood pressure, mmHg	143.31 ± 23.71	140.5 ± 22.9	146.0 ± 24.3	0.02 *
Diastolic blood pressure, mmHg	74.14 ± 11.21	75.2 ± 11.3	73.1 ± 11.0	0.07
eGFR, mL/min/1.73 m^2^	26.34 ± 15.79	28.1 ± 16.6	24.8 ± 15.0	0.02 *

Abbreviations: CKD, chronic kidney disease; BMI, body mass index; eGFR, estimated glomerular filtration rate; HbA1c, glycated hemoglobin. Note: Patients with CKD Stages 1 and 2 (eGFR ≥ 60 mL/min/1.73 m^2^) were excluded from this study. Data presented are mean ± standard deviation or frequency (percentage), as appropriate. *p*-value was based on chi-square or *t*-test, as appropriate. * indicates statistically significant.

**Table 3:** Risk factors associated with any ocular disease, AMD, and DR in multivariable regression models.

	Any Ocular Disease		AMD		DR	
Risk Factors	OR (95% CI)	*p*	OR (95% CI)	*p*	OR (95% CI)	*p*
Age, per year increase	1.05 (1.02–1.07)	<0.001 *	1.05 (1.02–1.07)	<0.001 *	0.95 (0.92–0.98)	<0.001 *
Sex, female	0.83 (0.53–1.28)	0.4	0.82 (0.54–1.26)	0.4	1.28 (0.67–2.46)	0.5
Diabetes, yes	2.52 (1.61–3.95)	<0.001 *	2.53 (1.62–3.94)	<0.001 *	-	-
Hypertension, yes	1.03 (0.5–2.15)	0.9	1.02 (0.49–2.11)	1	0.32 (0.08–1.31)	0.1
BMI, per unit increase	0.96 (0.92–1.01)	0.1	0.96 (0.92–1)	0.1	0.94 (0.88–1)	0.1
HbA1c %	-	-	-	-	1.24 (1.02–1.51)	<0.001 *

Multivariable logistic regression models were adjusted for age, sex, diabetes, hypertension, and BMI. The DR model (in those with diabetes) was additionally adjusted for HbA1c. Abbreviations: AMD, age-related macular degeneration; CKD, chronic kidney disease; CI, confidence interval; DR, diabetic retinopathy; OR, odd ratio. * indicates statistical significance.

**Table 4:** Summary of studies reporting ocular findings in patients with CKD.

Author, Year	Study Design	Population	Key Findings
Deva, 2011 [26]	Cross-sectional	Patients with CKD stages 3 to 5	Advanced CKD patients had a higher prevalence of vision-threatening retinal abnormalities, including moderate to severe microvascular retinopathy (39%), DR (28%), and macular degeneration (7%). Renal failure was identified as an independent risk factor for these retinal conditions, with 7.3% of advanced CKD patients having undiagnosed sight-threatening abnormalities requiring urgent ophthalmologic care.
Evans and Rosner, 2005 [27]	Narrative review	Patients on chronic hemodialysis, United States	Chronic hemodialysis patients face a range of ocular complications, including glaucoma, band keratopathy, cataracts, retinal detachment, macular leakage, retinal hemorrhage, optic neuropathy, and drug toxicity. Dialysis-induced changes may worsen ocular diseases.
Grunwald et al., 2012 [16]	Cross-sectional	Chronic Renal Insufficiency Cohort, United States	Greater severity of retinopathy and presence of hypertensive retinal vascular signs were significantly associated with lower eGFR.
Liew et al., 2008 [28]	Longitudinal	Blue Mountains cohort, Australia	Five-year incidence of early AMD was significantly higher in moderate CKD (17.5%) vs. no/mild CKD (3.9%). Moderate CKD was associated with increased risk of early AMD (OR: 3.2; 95% CI: 1.8–5.7, *p* < 0.0001); each SD decrease in eGFR doubled the AMD risk.
Mohan et al., 2024 [21]	Retrospective	CKD patients in a tertiary care centre, India	Retinal pathologies are highly prevalent among patients with CKD, with DR being the most frequently observed condition, followed by hypertensive retinopathy
Wong et al., 2016 [17]	Population-based cohort	Singapore Epidemiology of Eye Diseases	CKD patients had a higher prevalence of VI and ocular disease; CKD is associated with increased odds of VI, cataracts, retinopathy, and DR.

Abbreviations: AMD, age-related macular degeneration; CKD, chronic kidney disease; CI, confidence interval; DR, diabetic retinopathy; OR, odd ratio; VI, visual impairment.

## Data Availability

As the study involved human participants, the data cannot be made freely available in the manuscript, the supplemental files, or a public repository due to ethical restrictions. Nevertheless, the data are available from the Singapore Eye Research Institutional Ethics Committee for researchers who meet the criteria for access to confidential data. Interested researchers may request access by contacting the Singapore Eye Research Institute at: seri@seri.com.sg.

## Abbreviations

The following abbreviations are used in this manuscript:

AMD	Age-related macular degeneration
BMI	Body mass index
BP	Blood pressure
CKD	Chronic kidney disease
CRAO	Central retinal artery occlusion
CRVO	Central retinal vein occlusion
DR	Diabetic retinopathy
eGFR	Estimated glomerular filtration rate
ERM	Epiretinal membrane
HbA1c	Glycated hemoglobin
IRB	Institutional review board
IRED	Retinal imaging in renal disease
NPDR	Non-proliferative diabetic retinopathy
NUH	National University Hospital
PDR	Proliferative diabetic retinopathy
SERI	Singapore Eye Research Institute
UACR	Urine albumin-to-creatinine ratio
VI	Vision impairment
