# Correction: Liem et al. High Ocular Disease Burden and Increased Referral Needs in Patients with Chronic Kidney Disease: A Step Toward Personalized Care. *J. Pers. Med.* 2025, *15*, 204

**DOI:** 10.3390/jpm15100494

**Published:** 2025-10-15

**Authors:** Yulia Liem, Pavitra Thyagarajan, Miao Li Chee, Cynthia Ciwei Lim, Boon Wee Teo, Charumathi Sabanayagam

**Affiliations:** 1 Singapore Eye Research Institute, Singapore National Eye Centre, Singapore 169856, Singapore; yulia.liem@seri.com.sg (Y.L.); e0498207@u.nus.edu (P.T.); chee.miao.li@seri.com.sg (M.L.C.); 2 Department of Renal Medicine, Singapore General Hospital, Singapore 169856, Singapore; cynthia.lim.c.w@singhealth.com.sg; 3 Department of Medicine, Yong Loo Lin School of Medicine, National University of Singapore, Singapore 117597, Singapore; mdctbw@nus.edu.sg; 4 Division of Nephrology, Department of Medicine, University Medicine Cluster, National University Health System, Singapore 119228, Singapore; 5 Ophthalmology and Visual Sciences Academic Clinical Programme, Duke-NUS Medical School, Singapore 169857, Singapore

**Error in Figure and Table**

In the original publication [1], there was a mistake in Table 1 and Figure 1 as published. The correction pertains to the clarification of data reported in the section regarding annual referrals (Table 1). In Figure 1, there was a mislabeling in the graph legend. The label “Follow-up without referral” should be corrected to “No referral”, as the original label inaccurately represented the data. The corrected Table 1 and Figure 1 appears below.

**Text Correction**

A correction has been made to Section 3, Results, paragraphs number 2 and number 3. In Table 1, the original total of 341 includes those who were recommended annual rescreening as well those with no mention of annual rescreening. We did not differentiate these two groups in our accepted version. We have now clarified and revised the numbers to 154 (Routine annual) in Table 1. We also have revised the affected sentences pertaining to the changes as follows:

Of the 528 participants, 187 (35%) required referral for ocular conditions and 154 (29%) were recommended for annual rescreening for conditions such as mild NPDR or early AMD, as shown in Table 1. The remaining 187 (35%) participants did not require any referral. Among those referred, six received urgent referrals for conditions such as CRAO, macular hole, PDR, retinal detachment, and retinal emboli. An additional 30 participants were referred within 1–2 weeks (semi-urgent), the majority of which were for PDR at 63% (n = 19), followed by late AMD at 20% (n = 6). Within the three-month referral group, 29% (n = 151) had fast-track referral. Among these, two cases had ungradable images, while 17 cases were referred for monitoring due to “treated stable DR”. When stratified into CKD stages, referral rates for <3 months (n = 161), referral rates increased with CKD progression from, 43% in Stage G3, to 58% in G4, and 72% in G5.

When stratified by diabetes status and follow-up status when available (n = 418) (Figure 1), 67.4% of the 251 participants with diabetes were on annual follow-up. However, 51% of those referred to annual follow-up had to be fast-track referred for conditions like referable DR, glaucoma/glaucoma suspect, ERM, AMD, etc. Of the 167 non-diabetic participants, 31.1% were on follow-up, among whom 7.8% required fast-track referral. Of the non-diabetic participants not on eye follow-up (69% in total), 11% required fast-track referral. Six of the non-diabetic and 12 of the diabetic patients required urgent referral within one week for conditions such as pseudo-holes, impending occlusions, disc swelling, etc.

The authors state that the scientific conclusions are unaffected. This correction was approved by the Academic Editor. The original publication has also been updated.

## Figures and Tables

**Figure 1:**
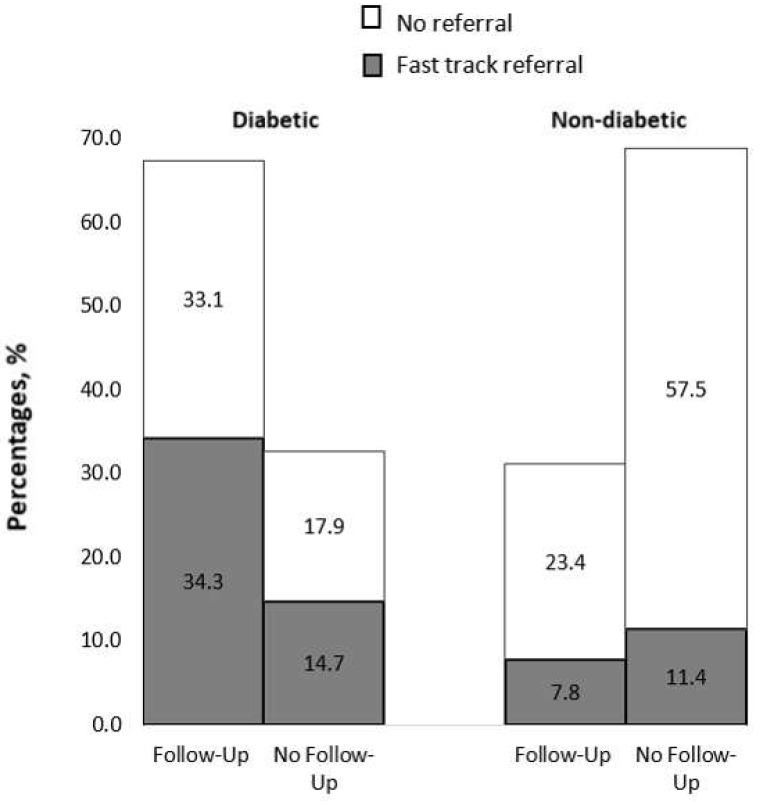
Percentages of routine monitoring among CKD patients, categorized by diabetic (n = 251) and non-diabetic (n = 167) status.

**Table 1:** Classification of referrals based on urgency and ocular conditions (n = 341).

Referral	Number of Cases	Causes
Urgent (immediate)	6	CRAO, macular hole, PDR, retinal detachment, retinal emboli
Semi-urgent (1–2 weeks)	30	Central Serous Retinopathy, collaterals with impending occlusion/disc collaterals, CRVO, late AMD, PDR with maculopathy, pseudohole due to ERM
Fast-track (1–3 months)	151	Cataracts, glaucoma suspect, late AMD, maculopathy, mild NPDR with maculopathy, moderate NPDR, ungradable, treated stable DR
Routine (Annually)	154	Early AMD, mild NPDR, myopic degeneration, presence of asteroid hyalosis

Abbreviations: AMD: age-related macular degeneration; CRAO: central retinal artery occlusion; CRVO: central retinal vein occlusion; ERM: epiretinal membrane; NPDR: non-proliferative diabetic retinopathy; PDR: proliferative diabetic retinopathy.
